# Supplementary material for: Are carbon emissions trading and green financial instruments synergistic? -Comprehensive quantitative research based on content analysis
Source: PLoS One. 2024 Mar 7;19(3):e0298601. doi: 10.1371/journal.pone.0298601 (PMC10919601; doi:10.1371/journal.pone.0298601)
Supplement: S1 Data — (ZIP) [file pone.0298601.s001.zip › Data/地级市Z_F工作报告.zip!/─■╧─/╓╨╬└╩╨/└┤╫╘╣1⁄2╓┌║┼í╛┬φ┐╦╩2╛▌═°í┐_2013.docx]

各位代表：

我代表市人民政府，向大会报告过去五年的工作，对未来五年目标和明年的工作提出建议，请予审议，并请政协委员和列席人员提出意见。

过去五年工作回顾

第二届市人民政府履职以来，走过了五年非凡的历程。在自治区党委、政府和市委的坚强领导下，在市人大、政协的有力监督支持下，我们坚持以邓小平理论、“三个代表”重要思想和科学发展观为指导，全面认真贯彻落实自治区第十次、第十一次党代会和市第二次、第三次党代会精神，深入学习宣传贯彻党的十八大精神，解放思想，开拓创新，求真务实，拼搏进取，积极应对复杂多变形势，克服种种困难和不利因素，建设和谐富裕新中卫取得了历史性重大成就。

——经济实力迈上一个大台阶。五年地区生产总值、工业增加值和地方公共财政预算收入均实现翻番；地区生产总值年均增长13.3%，实现了追赶跨越，成为全区经济增长最快的城市。中卫已经跃升为宁夏经济社会发展的重要增长极。

——城乡面貌发生历史性变化。五年城市建成区扩大了3倍，73%的农村土坯房换成了砖瓦房，城乡基础设施基本健全，城市绿地面积从1200亩增加到2.9万亩，湖泊水系面积从900亩增加到2.2万亩，街道干净，植被青葱，城乡环境质量根本改善。

——人民生活水平显著提高。五年城镇居民人均可支配收入由9772元提高到17960元，农民人均纯收入由3124元提高到5850元；建立健全了社会保障救助体系，新增就业岗位4.2万个；贫困人口减少13万人。

——教育取得重大进步。全面实现“三免一补”，实施校舍安全改造工程，创建教育强县区，建设国家级职业教育学校，这些是我市教育发展史上的里程碑。全市整体教育水平得到较大提升。

——社会建设得到加强。改革优化沙坡头区行政体制，完善行政机构与功能，加强乡镇和社区管理，取得了明显效果。

——民主法制建设取得新进步。依法行政扎实推进，保障人民权益和维护社会公平正义得到加强。

——生态建设取得重要成果。构建生态安全新格局，防沙治沙用沙256平方公里，实施了美利林纸一体化造林和南华山水源涵养基地造林工程，恢复湿地面积6.2万亩，实现了沙漠林纸一体化、沙漠工业园区化、沙漠旅游产业化。

——政府自身建设全面加强。健全科学决策机制和民主议事程序，召开常务会议168次，议决议题1533个；制定了69项创新制度和148项规范制度，建设职能科学、结构优化、廉洁高效、人民满意的服务型政府取得了阶段性成效。

各位代表，过去五年是中卫发展进程中抢抓机遇、乘势而上的五年，是我们经受住各种困难和风险考验的五年，是我们夺取建设和谐富裕新中卫、全面建成小康社会新的更大胜利的五年。即将过去的2012年，面对经济环境加压、发展竞争加剧、社会管理难度加大的严峻考验，我们见事早、行动快，谋大事、求实效，全市经济保持较快增长，社会保持和谐稳定。预计全年实现地区生产总值253亿元，比上年增长12%；完成固定资产投资245亿元，增长26%；实现地方公共财政预算收入14.5亿元，增长16.1%；规模以上工业增加值达到64亿元，增长16%；社会消费品零售总额达到44.5亿元，增长16%；城镇居民人均可支配收入17960元，增长13.2%；农民人均纯收入5850元，增长13%。圆满完成了市二届人大六次会议确定的各项目标任务。

五年来，我们付出了巨大努力，做了大量工作。

一、不断优化投资发展环境，工业经济健康高速增长

着力建成产业转移承接地。始终坚持工业化主导、城市化带动，充分发挥区位、交通、土地、水利不可复制的组合优势，在戈壁荒滩和低丘缓坡地上建成中卫、中宁工业园区和海原工业物流园区，以及镇罗金鑫园、常乐陶瓷园等6个专业园区，成为中卫承接产业转移的高地和招商引资企业的福地。入园企业228家，建成投产169家，全部达产达效后可实现工业增加值320亿元。形成了新能源、新材料、冶金、化工、造纸、农副产品深加工六个产业板块，工业增加值五年增长2.1倍，年均增长18.4%，工业对地区生产总值的贡献率达到54.3%。中卫市利用荒山荒漠发展工业得到国土资源部充分肯定，被列入全国首批低丘缓坡荒滩未利用地试验区。

制定发展规划和产业扶持政策。编制了《中卫市发展战略规划研究报告》，完成了《中卫市产业发展规划》、《中卫工业园区（东扩区）总体规划》。积极应对严峻经济形势，出台了《工业与固定资产投资保增长18条措施》等，累计投入扶持资金22.6亿元。这些救企政策使工业经济在多次出现经济下行压力下实现了平稳较快增长。

招商引资取得显著成果。抢抓国家产业转移和西部大开发重大机遇，制定《招商引资优惠政策及奖励办法》，建立市级领导包抓重点招商项目制度和常务会议督办机制，储备项目抓开工、开工项目抓进度、在建项目抓投产、投产项目抓达效，引进项目703个，到位资金596亿元，年均增长56%，是前五年的7倍。新疆广汇能源深加工、重庆紫光蛋氨酸等产值过百亿的重大项目落地开工。锦宁一期42万吨铝镁合金、宁钢一期70万吨特钢、银阳一期5000吨多晶硅等一批大项目好项目建成投产，工业效益大幅提升。中卫市被评为“中国投资环境十佳城市”和“全国十大最具投资潜力城市”。

全面促进资源节约与环境保护。高度重视发展循环经济，促进生产、流通、消费过程的减量化、再利用、资源化。加快建设中卫、中宁两大循环经济示范区，累计淘汰钢铁、水泥、电石、铁合金等行业落后产能265万吨，年节约标准煤89万吨，初步实现了园区废水循环使用、尾气余热回收、废渣资源化利用。认真落实结构减排、工程减排、管理减排措施，全面完成了“十一五”减排任务，中卫城市环境空气质量持续好转，优良天数达到316天以上，城市饮用水源地水质稳定在三类良好水质。

二、不断加大“三农”投入，现代农业持续发展

落实各项富民惠农政策。把“三农”工作摆在重中之重，累计投入“三农”资金58亿元，占财政预算支出的19.4%，年均增长30.1%。发放各种农业补贴6.8亿元，惠及23.8万户90.6万人；兑付退耕还林、退牧还草补贴5.8亿元，全部以“一卡通”发放，极大地调动了农民的生产积极性。2012年全市粮食总产突破60万吨，实现了连续八年增长。

加大农业基础设施投入。累计投资41.8亿元，实施兴仁综合供水、南北干渠节水改造等项目410个，砌护渠道5000公里，治理水土流失1043平方公里，改造中低产田50万亩。投资11.6亿元，完成沙坡头区宣和、兴仁，中宁红梧山、撒不拉滩，海原高崖等土地开发整治项目，新增耕地27.7万亩，基本农田达到292万亩，连续五年完成基本农田保护目标任务。

大力扶持优势特色农业。农业特色优势产业已经成为拉动农民增收的支柱产业。硒砂瓜种植面积达到92.3万亩，设施蔬菜、枸杞种植面积分别达到30.1万亩、36万亩；优质饲草面积巩固在115万亩，经果林面积达到102万亩；马铃薯、优质米、小茴香种植面积分别达到109.5万亩、14.5万亩和12.1万亩。建成20万亩硒砂瓜品牌保护核心区、2.7万亩供港蔬菜基地、20万亩脱毒种薯基地。建设4万亩绿色有机枸杞基地、33个标准化养殖园区。中宁枸杞、香山硒砂瓜、设施蔬菜成为全国绿色食品原料标准化生产基地。全市各类农产品加工企业发展到1210家，农民专业合作组织和农产品流通协会达到584个。出台了《中宁枸杞品质品牌保护办法》、《硒砂瓜品牌保护方案》，“宁夏红”、“中宁枸杞”、“香山硒砂瓜”荣膺中国驰名商标，品牌效应凸显，市场竞争力显著提升。

三、不断推进城乡一体化进程，人居环境质量显著提升

完成城市总体规划和专项规划修编。修编了《中卫城市总体规划（2011—2030）》，编制了城市综合交通、给排水、供电、供热、燃气等10余项基础设施专项规划，城市中心区规划面积由51.5平方公里调整到64.7平方公里。完成了城市重点建设区域控制性详细规划和重点项目建设规划，为建设美丽中卫绘就了美好蓝图。

城市建设取得重大成果。投资191亿元，新建、续建城市基础设施项目567个。建成中卫城市新区、中宁新区和海原工业物流园区，完成中卫、中宁工业园区道路框架等基础设施。新建改建中营高速等公路2854公里，全市公路通车里程从2007年的4625公里增加到6502公里，全市公路密度每百平方公里达到37公里。新建市区南北滨河路、机场路等城市道路300公里。建成沙坡头机场，东、西两座跨铁路大桥，敷设市政管网495公里，建成5座供热站和1座供水厂、1座污水处理厂。城市集中供热率达到90.3%，垃圾和污水实现了集中无害化处理。房地产业繁荣发展，新建商品房420万平方米，建成中卫、中宁、海原等124幢98.4万平方米行政办公大楼，建成“五馆一中心”等一批公共文化建筑。开工建设“大河之舞”主题文化公园、全民创业城等重点项目。宣和、镇罗、石空、三河等特色小城镇建设加快推进，全市城镇化率由25.3%上升到34%，提高了8.7个百分点。

实施城市绿化美化净化工程。以绿为美、以水为源、以净为荣、以人为本，全力打造生态中卫。做大做美“绿”文章。新增绿地2.8万亩，城市绿地率、绿化覆盖率分别达到36.6%和37.9%，分别提高了29.7和27.5个百分点。实施“美丽绿化”机制，植物年浇水量达到900毫米，使植被生长更快、更健康、更美丽。做足做秀“水”文章。建成市区景观水系和腾格里湿地公园、黄河公园、香山公园、应理湖及“五馆一中心”等湖泊水系。利用腾格里沙漠丰富的自然渗水资源，连通“六大水域”，注重水位、水质和流动性设计，使水域带给城市秀美与活力。做深做实“净”文章。实施“深度清洁”机制，建立机械深度洗扫和人工即时保洁环卫新模式，街道广场等公共场所地面每平方米浮尘不到5克，新区街道实现了无垃圾目标。启动城市“六创”活动，沙坡头区、中宁县跨入自治区园林城市、卫生城市行列。

大力推进农村环境综合整治。实施农村危房改造、农村基础设施建设和城乡环境综合整治“三大工程”，投资78.3亿元，建成“塞上农民新居”示范点71个，综合整治旧村庄374个，改造农房7.4万户。新修农村道路500公里，铺设供排水管道750公里，建设农村垃圾中转站18座、填埋场10个，配备垃圾清运车675辆、垃圾箱（桶）2.9万个。农村垃圾集中处理率从2007年的5%提高到85%，农村人居环境明显改观。

四、不断提升旅游产业战略地位，第三产业加快发展

旅游产业快速发展。全市共有旅游景区30个，五A级景区1家，三A级景区3家，旅行社7家，各类酒店39家，床位8000张，餐位38000多个，农家乐53家。全市累计接待游客830多万人次，旅游总收入52亿元。旅游基础设施日趋完善，产业链条逐步拉长，品牌形象快速提升，食、住、行、游、购、娱等六大要素协调配套的产业体系初步形成，旅游产业已成为我市具有鲜明特色和竞争力的优势特色产业之一。

确立了旅游优先发展战略。事实证明，旅游业能够带来巨大经济效益和社会效益，旅游业是中卫实现又好又快发展的关键性支柱产业。沙坡头景区是具有垄断性的世界级旅游资源，中卫旅游业完全能够依托沙坡头这个龙头景区做大做强，成为中卫重要的支柱性产业。我们得举全市之力，把沙坡头品牌做响做出影响力，把中卫打造成与桂林、张家界等齐名的旅游目的地城市，让沙坡头成为宁夏之最、中国之最、世界之最。

整合资源构建旅游支柱产业发展格局。编制了《中卫市城市旅游发展总体规划》、《黄河沙坡头大峡谷总体规划暨重要节点修建性详细规划》、《沙坡头核心区景观提升修建性规划》、《腾格里大酒店建设规划》、《滨河五星级酒店方案设计》、《沙坡头水镇规划》、《千年党项第一村概念性规划》、《沙漠生态园规划设计》等，为旅游业持续发展绘制了蓝图。整合沙坡头南北景区，组建了国有控股的沙坡头旅游景区公司和索道公司；召开旅游体制改革大会，成立宁夏沙坡头旅游产业集团、沙坡头旅游投资控股公司、旅游规划委员会、旅游专家咨询委员会，有效解决了一些影响和制约发展的体制障碍和深层次矛盾，发展活力进一步增强，中卫旅游迈上了科学发展、创新发展、跨越发展轨道。

提升旅游景区接待能力和影响力。开工建设了黄河沙坡头大峡谷、千年党项民俗村等旅游项目，建成金沙岛旅游休闲度假区、沙坡头景区仪门、游客中心，南北景区实现了立体交通，景区接待服务能力大幅提升。增开了旅游专列，试开了重庆、杭州等重要客源地市场旅游包机，举办了沙坡头首届中国超卡大赛，电视剧《风雨沙坡头》获中国文化旅游发展贡献奖。依托沙坡头品牌，采取“政策激励促销、利用载体促销、举办活动促销、精品线路促销、借助媒介促销”等方式，促使来卫游客以50%的速度递增。在全国范围内快速提高了沙坡头的知名度、美誉度，掀起了中卫旅游“大营销”的高潮。

加快繁荣现代服务业。“外煤进宁”铁路通道取得实质性进展，甘武二线及迎水桥延伸段共236公里的铁路项目获批，将于明年开工建设。建成中宁东站战略装车点，全市货物吞吐量由1500万吨增加到了2600万吨。建成世和新天地、朝阳百货、中宁枸杞国际交易中心、海原农副产品批发市场等一批商贸项目；开工建设城市商贸综合体、四季鲜农产品批发市场等现代服务业设施；引进了苏宁电器、华润万家、江苏横店数字影院等知名商家入驻中卫。成立了中卫农村商业银行，新增金融机构17家，贷款余额由83.8亿元增加到了245亿元，年均增长23.9%。五年社会消费品零售总额增长了1.4倍。

五、不断加强社会建设，民生民利显著改善

切实保障和改善民生。扎实推进民生计划实施。五年累计办理民生实事57件。其中在全国率先开通了农村公交，实施了沙坡头区居民饮水水质改造工程和兴仁综合供水工程，实现了城乡居民饮水安全。实施贫困地区农村中小学生营养改善计划。新建幼儿园10所，幼儿入园率从39.4%提高到49%。建立农民工新市民制度，1.1万名务工人员成为城镇新市民。进一步完善社会保障救助制度。统筹城乡居民社会养老保险、医疗保险覆盖面不断扩大，参保率分别达到77%和95.5%。在全国率先建立了重特大病医疗救助制度、重度残疾人生活津贴制度和农村养老补贴等救助制度，重特大病患者、农村老人、重度残疾人和孤儿生活就医有了保障。成立市公益慈善基金会，募集资金8966万元，中卫被评为“全国六星级慈善城市”。建立以公租房、廉租房为主的多层次住房保障体系，建成保障性住房2.54万套142.7万平方米，保障率达到22%，居全区首位，人民群众享受到改革发展的最新成果。

推进城乡教育均衡发展。五年累计投入资金51.6亿元，比前五年增长了2.6倍，占地方公共财政预算支出的比重达到17.2%。完成375所学校73万平方米的校安工程，新建中卫中学、六中、八小、特殊教育学校、中宁一中、海原四中等50所中小学校和中卫职业技术学校，开工建设中卫九小，基本解决了市区义务教育“大班额”问题。实行普通高中困难家庭学生“三免”政策，促进公共教育资源共享，高中毛入学率五年提高17.7个百分点。购置了40辆安全校车，在全区率先实现了校车的安全运营和即时监管。

不断繁荣发展文体事业。实施文化惠民工程，建成并开放使用市体育馆、文化馆、图书馆、文体娱乐馆，实现了农家书屋、农村直播卫星服务“户户通”全覆盖。成功举办了奥运圣火传递、环青海湖国际公路自行车赛中卫赛段、CBA男篮四强邀请赛和全市第一届中学生田径运动会，群众精神文化生活日益丰富。实施文化旅游“六个一”工程，组建了中卫市歌舞团，《回乡婚礼》首次走出国门赴阿拉伯国家交流演出，海原县被文化部命名为“中国民间剪纸文化艺术之乡”，中卫的知名度和美誉度大幅提升。

深化医药卫生体制改革。全面实施国家基本药物制度，实行药品零利润销售，有效地减轻了群众看病负担。公立医院改革迈出扎实步伐。创新医药卫生支付制度，提高乡镇卫生院、村卫生室、社区卫生服务站医保报销比例。实施药品电子化监管。开工建设市医疗中心，建成宁南中心医院、市中心血站和市县120急救中心。新建、改扩建乡镇卫生院、村卫生室和社区卫生服务站205个，人民群众享受到更加便捷的公共卫生服务。建立以抓山区人口、流动人口出生率为重点的计划生育四级包保监控责任机制，人口出生率突破性地降至12.6‰以内的低生育水平。建立餐饮卫生安全监管无遗漏新机制，全市连续五年未发生重大食品药品安全事故。

加快生态移民脱贫步伐。建成30个生态移民项目区，搬迁安置移民1.5万户6.6万人。着力培育发展肉牛养殖等移民后续产业，组建了乐农生态移民产业发展小额贷款公司，发放养牛贷款1500万元，建成肉牛养殖示范村4个，入栏基础母牛1462头，发展经果林3000亩。大力实施生态移民“5231”就业计划，职业技能培训1825人，中卫、中宁工业园区吸纳移民就业3000人，确保移民“搬得出、稳得住、逐步致富奔小康”。

六、不断推进体制机制改革，政府效能明显提升

创新经济管理制度，提高推动发展能力。建立政府偿贷基金制度，搭建了政府信用平台，五年筹集偿贷基金16.9亿元，撬动银行贷款45.8亿元，使融资有了信用保障、偿贷有了资金保障、发展有了投资保障。出台《支持中小微企业繁荣发展的20条政策》，破解中小微企业发展难题，行政审批（收费）事项从222项减到124项，为中小微企业营造了良好的发展环境。

创新行政管理制度，提高政府行政效能。设立市政务服务中心，实行行政审批服务事项“一站限时”办结制，五年累计办结20.1万件，按时办结率100%。实施国家工作人员法律与专业三年培训规划和手机考勤制度，在全区率先开通了移动远程视频办公系统，推行“五定”工作法，实行市长督办副市长、副市长督办部门工作机制，强力推进重点工作落实。

创新廉政建设制度，提高预防腐败能力。充分发挥制度在廉政建设中的关键作用，在全区率先实行了政府投资项目造价预算与建设管理分离制度和代建项目造价包干制，有效预防了工程建设领域的腐败。出台了《政府投资项目单位工程预算标准》，从八大类28项166个具体项目逐项明确了单位预算标准，使政府投资项目有规可依、有价可控，大幅节约建设资金。出台了政府廉政效能建设九项制度和进一步营造风清气正发展环境活动九项制度，优化了发展环境，中卫市政风行风评议连续四年名列五市第一。

创新社会管理制度，提高依法治市能力。设立沙坡头区管理委员会，填补了沙坡头区无行政机构的空白，加强了沙坡头区社会管理。创新社区管理模式，实施区直管社区和无土地村改居试点工作。实施创建“平安中卫”十大亮剑行动，改革警务模式，建立交巡警合一的道路巡防制度，整治交通黑点520处。建立安全生产在线智能视频监控平台，实现重点场所24小时在线监控，安全生产事故年死亡人数从98人下降到58人，下降比例全区第一。落实信访“五联动”机制和“三调联动”综合调解机制，及时化解矛盾纠纷，信访总量下降3.34%。依法加强宗教事务管理，全市宗教和顺、民族和睦、社会和谐。

全民国防教育、双拥共建、民兵预备役、防震减灾、人民防空、外事侨务、市志档案、邮政通信、气象服务等各项事业都取得了新的业绩。

各位代表，过去五年所取得的成绩，归功于自治区党委、政府和市委的正确领导，得益于历届政府打下的良好基础，有赖于人大、政协和社会各界的鼎力支持，源自于全市人民的艰苦奋斗。在此，我代表市人民政府，向全市各行各业辛勤劳动的建设者，向各位人大代表、政协委员，各民主党派、工商联、各人民团体，向中央和自治区驻卫单位，驻卫部队、武警官兵，向所有关心、支持和参与中卫发展的各界人士、企业家们，表示崇高的敬意和衷心的感谢！

在充分肯定成绩的同时，我们也清醒地认识到，中卫经济社会发展还存在一些矛盾和问题：一是经济发展尚处于起步阶段，总量小、效益低、结构单一；二是城市与农村、灌区与山区发展不平衡，扶贫攻坚任务重；三是事关群众切身利益的问题需进一步解决，维护社会稳定的任务还很艰巨；四是保障科学发展的体制机制不够完善，各类人才匮乏，打造服务型政府任重而道远。对此，我们必须高度重视，采取有力措施，切实加以解决。

各位代表，五年的前进征程步履坚实，五年的发展成果来之不易，五年的创业体会弥足珍贵。

第一，必须始终坚持科学发展。要善谋战略性举措，提升发展战略，做正确的事，正确地做事；要切实把“四大战略”落实到中期规划中、年度项目中、当前行动中；只有方向正确，措施得力，我们才能在既定时间完成既定目标。

第二，必须坚定不移地推进改革开放。要着力推进旅游、医药、教育、财政和政府投资、项目建设、城市管理、节水模式等体制机制改革；要善于发现制约因素，勇于破除体制机制障碍，以此持续不断释放出新空间、新活力、新机遇，带来新气象、新变化、新发展。

第三，必须提供良好公共服务。良好公共服务是开放的前提，从公共利益出发，是服务型政府的根本标志。我们必须强化公共服务职能，逐步建立起符合中卫实际、切实有效的公共服务体系；进一步简政放权，加快从管理型政府向服务型政府转变，培养一支高效、勤奋的公职人员队伍。

第四，必须高度重视培育区域优势。追求经济发展必须打造区域优势，推进发展不仅需要区位优势和丰富资源等硬实力，更需要一系列与社会主义市场经济相适应的制度安排、激励机制和创新精神等软实力；只有坚持创新引领，研究制定一系列好政策、好机制、好制度，才能打造一个充满活力、具有巨大吸引力、能够不断产生和造就千千万万创业者的区域优势。

第五，必须坚持依法行政。遵纪守法、遵规守则是政府一切工作的根本原则，依法行政事关百姓利益、事关公平正义、事关行政效能、事关党风廉政。我们必须在职权内履行职责，加强行政规范性文件制定，不断把科学的办法标准化，有效的管理制度化，努力建设法治政府，让权力在阳光下运行，让人民利益不受侵害！

未来五年目标及2013年主要任务

新一届政府任期的五年，是我市深入贯彻落实党的十八大精神、全面实现“十二五”规划、开启“十三五”建设新征程的五年。自治区大力实施沿黄经济区和扶贫攻坚两大战略、中央支持宁夏“两区”建设，为我市跨越发展带来了重大机遇；新一轮产业转移将由资源主导、劳动密集加速向资本集约、技术集成转变，为我市承接东中部产业转移、推进产业转型升级提供了项目支撑；设市以来，基础设施改善、产业体系建立、社会事业进步等发展成果，为我市跨越发展奠定了雄厚基础。我们必须牢牢抓住新机遇，积极应对新挑战，以奋发有为的士气、志在必得的勇气、创先争优的锐气，因势利导，顺势而为，乘势而上，将中卫放在全国、全区发展大局中去思考、去谋划、去定位，努力在新一轮区域比拼中把握先机、争取主动，开创中卫下一个五年的辉煌。

未来五年的主要预期目标是：全市地区生产总值年均增长12%；全社会固定资产投资年均增长25%；地方公共财政预算收入年均增长15%；城镇居民人均可支配收入和农民人均纯收入分别增长12%和12%；人口自然增长率控制在9.5‰以内；万元地区生产总值综合能耗降低10%；全市森林覆盖率力争达到16%。

2013年是新一届政府的开局之年。做好政府工作，事关中卫全面建成小康社会的大局，使命光荣、任务艰巨。市委对政府工作的总体要求是：以科学发展观为指导，认真贯彻落实党的十八大、自治区第十一次党代会和市委三届三次全会精神，以建设和谐富裕新中卫为目标，围绕“五位一体”总体布局，加快推动“四大战略”，努力建设“四地”，深化改革、提高内外开放水平，加快经济结构调整、促进经济又好又快发展，创新社会管理、改善民生，建设生态文明、打造美丽中卫，加强民主法制建设、提升政府效能，为全面建成小康社会迈出坚实一步。

主要预期目标是：地区生产总值增长12%左右；地方公共财政预算收入增长15%；固定资产投资增长26%左右；工业增加值增长16%；城镇居民人均可支配收入增长12%；农民人均纯收入增长12%；万元地区生产总值综合能耗降低2.86%；人口自然增长率控制在10.05‰以内；城镇登记失业率控制在4.5%以内。

完成上述目标，我们必须做好以下工作：

一、抢抓“两区”建设新机遇，着力深化改革开放

紧紧抓住“两区”建设重大机遇，创新体制机制、深化改革开放，在优先发展旅游产业、打造“宁东西区”、发展现代物流、优化发展环境上实现新突破。

打造国际化沙漠旅游目的地。坚持以国际化视野推动旅游体制机制创新，创设旅游开发区，打造旅游“产业引擎”和“创新平台”。发挥沙坡头旅游产业集团、投资控股公司、景区公司的龙头带动作用，探索发行企业债券，启动沙坡头旅游上市进程。按照“城旅一体化、城市景区化”要求，围绕旅游规划建设城市、完善基础设施、提升城市和景区接待能力，完善各旅游要素的配套衔接，努力形成旅游的政策洼地、人才高地、投资福地和创新天地。

打造能源加工转化和战略储备基地。加快“外煤进宁”通道建设，开工建设甘武二线及迎水桥延伸段、广申大道及跨铁路立交桥，提升综合承载能力。围绕“外煤进宁”和西气东输，编制《区域能源化工储备基地及资源深加工产业转移承接规划》，优化生产、运输、储备布局，联合宁东管委会，重新规划中卫甘塘能源化工基地，启动“宁东西区”建设，主动承接国内外产业转移，重点开发煤和天然气深加工项目，争取建设成为区域性大型综合能源化工加工及储备基地，形成宁夏能源化工产业东西协同联动发展态势，大幅提升中卫战略地位。

打造新欧亚大陆桥现代物流中转地。依托中卫在全国路网布局中独特的区位优势，积极争取国家、自治区支持，将中卫纳入到新欧亚大陆桥和渝新欧大通道的大格局中，加快包兰线扩能改造、西线高速公路中卫延伸段、海原工业物流园区至县城快速通道等重大项目的推进，不断完善交通基础设施。积极引进大型专业机构和物流集团，重点推进中卫甘塘煤炭物流园区、中卫综合物流园区、中宁物流园区、海原工业物流园区建设，力争将中卫建设成为全国西能东运集散地、新欧亚大陆桥重要的物流中转地。

打造环节最少、程序最简、费用最低的一流服务高地。依托“试验区”建设，建立政府、民间、企业多层次交流的开放合作机制。持续推进行政审批制度改革，减少行政审批事项，规范行政审批程序，形成良好的政务服务和法治环境。建立吸引人才、留住人才、使用人才的人才培养引进机制。落实支持中小微企业繁荣发展的20条政策等措施，为投资者创造更加便捷、更加有利的发展条件，激活各种发展要素，盘活各类优势资源，为中卫经济社会发展注入新活力。

二、转变经济发展方式，着力推动经济又好又快发展

适应国内经济形势新变化，加快转变经济发展方式，促进旅游和城市深度融合、工业和农业相互协调，加快推进旅游国际化、新型工业化、农业现代化、城乡一体化进程。

优先发展旅游产业。加快建设沙坡头旅游产业示范区，积极争取批复建设国家级旅游开发区。编制完成《中卫旅游总体规划》，创新旅游产业投融资体制，成立中卫旅游贷款担保中心，设立2000万元旅游发展专项基金，年递增20%。开工建设沙坡头南岸文化创意休闲岛、沙坡头风情小镇等10个旅游重点项目，完成投资3.8亿元。加快推进沙漠王朝酒店、沙漠汽车主题公园等重大项目前期工作，力争旅游总收入达到21亿元，增长20%。以旅游业带动现代服务业，全市新增酒店10家床位2000张，商贸流通企业达到1650家。加快金融机构建设，成立石嘴山银行中卫分行，改制组建中宁农村商业银行，全市金融机构贷款余额达到300亿元，增长25%，力争服务业占地区生产总值比重达到40%以上。

大力发展新型工业。开工建设中卫工业园区铁路专用线，加快完善东扩区基础设施，推进中卫、中宁工业园区一体化，形成350平方公里产业承接地。新建、续建工业重点项目56个，总投资950亿元，当年完成投资190亿元，重点抓好新疆广汇3×5万立方米液化天然气转运分销中心及煤化工、重庆紫光10万吨蛋氨酸、景津集团60万吨金属锰、银阳公司230兆瓦大型光伏电站及200兆瓦太阳能电池组件等重大项目。突出产业招商和项目招商，加快引进战略投资者，完成合同签约项目30个，招商引资到位资金260亿元。力促沙漠光伏产业园上海航天等12家企业500兆瓦光伏电站、宁钢集团320万吨特钢等19个项目落地开工。

稳步发展现代农业。加快建设沙坡头南北干渠改造、南山台扬水泵站更新改造等工程。实施20万亩高效节水现代农业示范项目，年内建成5万亩；规划建设20万亩有机硒砂瓜生产基地、20万亩绿色有机枸杞示范基地、20万亩高端葡萄酒产业园区、10万亩高垄管灌马铃薯基地、1万亩质数水稻基地。启动建设生态移民区4万头高端肉牛养殖园区，年内入园2万头；规划建设20万头奶牛产业园区，年内存栏2万头，力促农业增效农民增收。

加快推进城乡一体化。实施城市街巷改造、深度保洁、绿化亮化、秩序建设、精品开发“五大战役”和“六创”活动。完成街巷改造26条、城市棚户区改造986户。采用机械清扫、人工保洁相结合模式，确保市区环境卫生合格率达到100%。对主要街景、公园广场、地标建筑实行多组合多层次绿化亮化美化。规范整顿城市秩序，加强运输车辆防漏防渗防撒“三监管”，实行门前“五包”，做到有序行车、按位停车，美化街面广告、合理布设摊位。精心打造广汇综合生活服务基地、城市文化广场等一批城市精品项目。实施农村危房改造、垃圾收处、村容村貌改善、农村消防体系和天然气进农户“五大工程”。新建“塞上农民新居”示范点6个，综合整治旧村庄63个，完成危房改造9500户，完善垃圾集中清运处理机制，农村垃圾集中处理率达到90%。组建10个乡镇消防队、消防警务室，推广天然气进农户，年内完成2000户。加快中宁县城和海原大县城及兴仁、石空、三河等特色小城镇建设，进一步改革户籍管理，加快推进农民变市民步伐。

三、加强民主法制建设，着力促进社会公平正义

加快推进民主法制建设，努力建设为民务实、廉洁高效的服务型政府，对人民负责，受人民监督，为人民造福，让人民满意。

推进民主法制进程。健全决策机制和民主议事程序，做到科学民主依法决策，使各项决策符合法律法规，符合实际情况，符合人民意愿。自觉接受市人大及其常委会的法律监督、市政协的民主监督，坚持重大事项向人大报告、向政协通报，认真办理人大代表建议和政协委员提案。积极支持法院、检察院依法办案，加强同各民主党派、工商联、无党派人士的联系，倾听意见建议。加强基层政权建设，扩大农村（社区）民主选举，完善村（居）民代表会议等民主决策程序，健全村（居）务、财务公开等民主监督制度，实现自我教育、自我管理、自我服务为主的民主管理，推进村（居）民自治制度化、程序化、规范化。

大力推进依法行政。严格落实行政执法责任制，规范执法程序，做到有权必有责，用权受监督。加大政务公开力度，推进电子政务建设，增强政府工作的透明度。深化行政审批制度改革，简化程序，规范行为，提高行政效能。科学行使经济调节职能，善于运用发展规划、产业政策、财税政策、环境标准等手段管理经济事务，促进发展方式转变。积极推进行政效能监察，强化督察督办，确保政令畅通和各项重点工作落实到位。

加强和创新社会管理。实施“六五”普法，扎实开展法律援助工作。加强社会管理综合治理，深化平安创建活动，建立健全立体治安防控体系，依法打击违法犯罪活动，维护社会安定和谐。完善“大调解”工作机制，深入落实信访“五联动”机制和信访终结制度，设立信访民事巡回法庭，积极预防和化解社会矛盾。依法管理民族宗教事务，维护宗教领域和谐稳定。加强公共安全管理，健全防范突发事件、重大自然灾害及社会风险预警应急机制。加强食品药品安全监管，发挥安全生产视频即时监控平台作用，落实“三同时”、“三排查”和“一岗双责”制度，坚决杜绝重大安全事故发生，保障人民群众生命财产安全。

四、推动文化繁荣发展，着力丰富人民精神文化生活

加快发展文化事业和文化产业，满足人民群众多层次多样化文化需求，发挥文化引领风尚、教育人民、净化社会、推动发展的功能，全面增强社会凝聚力和创造力。

实施文化惠民工程。加大文化事业投入，加快完善公共文化服务体系，开工建设城市文化广场，建成中宁黄河体育中心。全免费开放使用市文化馆、图书馆，完成市博物馆布展工作。建成文化馆数字影厅4个，完成农村数字电影放映2000场次以上，组织开展大型广场文艺演出活动50场次，“文化下乡惠民工程”演出800场次。完善农家书屋功能，提升农村文化服务水平。举办全市第二届中学生运动会，广泛开展全民体育健身活动，丰富人民群众文化生活。

促进文化旅游融合发展。充分挖掘保护、开发利用南长滩党项民俗村、大麦地岩画、新旧石器遗址、海原环球大地震遗迹、中宁石空大佛寺等文化旅游资源，促进文化旅游深度融合。深化文化旅游“六个一”工程，编排具有中卫独特文化元素的精品剧目《神韵中卫》，依托中阿博览会平台，加强对外文化交流，积极承办国际国内重要文体赛事活动，扩大“沙坡头”旅游的国际知名度，努力打造中卫文化名片。

提高公民道德素质。深化民风建设“五大工程”，着力培育“遵纪守法、明礼诚信、奋进思变、淳朴向善、理性包容”的民风环境。加强社会公德、职业道德、家庭美德、个人品德教育，引导人们自觉履行法定义务、社会责任、家庭责任，营造劳动光荣、创造伟大的社会氛围。加强国防动员建设，促进军民融合式发展。加强和改进思想政治工作，深化群众性精神文明创建活动，广泛开展志愿服务，切实加强政务诚信、商务诚信、社会诚信和司法公信建设，打造诚信中卫。

五、加强社会建设，着力保障和改善民生

加强社会建设的根本目的是使经济发展成果真正惠及百姓、改善民生，新增财力70%用于改善民生，努力让人民群众学有所教、劳有所得、病有所医、老有所养、住有所居。

办好人民满意的教育。巩固教育强县区和基本普及高中阶段教育成果，均衡发展九年义务教育，加快发展职业教育，办好学前教育，支持特殊教育。建成中卫九小、中宁十小，完成海原回中迁建，改造建设10所幼儿园，实施50所14万平方米农村校舍改造工程，力促中卫职业技术学校升格为职业技术学院。全面实施素质教育，促进教育公平，合理配置教育资源，重点向农村、贫困、生态移民区倾斜，提高家庭困难学生资助水平，确保困难家庭和农民工子女享受同等教育，努力使孩子们接受“更好的教育”。

提高卫生医疗服务水平。建成市医疗中心、沙坡头区卫生监督所、海原县人民医院住院部和150所基层医疗卫生机构。建立健全县乡村三级医疗卫生服务网络和社区卫生服务体系。深化医药卫生体制改革，推行医药分离，设立5家政府医保药店，创新支付制度，乡镇、村（社区）医保报销比例分别提高到55%和65%。坚持计划生育和优生优育，提高出生人口素质，努力使人民群众享受到“更高水平的医疗卫生服务”。

完善社会保障救助体系。健全基本养老保险和医疗保险，完善老年人、残疾人、孤儿等困难群体的社会救助，切实解决好困难群体的生活救助，农民工工资“一卡通”覆盖面达85%以上。扎实推进全民创业，促进充分就业，培养小老板910名，创办小企业410个，创造新岗位4100个。建成福润苑等保障性住房8320套61万平方米，开工建设福兴苑等保障性住房9960套58万平方米，构建城市低收入家庭、农民工、失地农民、企业员工、新就业大学生多层次住房保障体系，努力使人民群众有“更可靠的社会保障”。

大力实施扶贫攻坚工程。认真落实《六盘山片区区域发展与扶贫攻坚规划》，扎实推进生态移民、产业扶贫和基础设施建设力度，年内减少农村贫困人口2.8万人。开工建设沙坡头区龙湖二期、敬农三期，中宁劳务移民区和海原七营、山门二期等生态移民工程，建成移民住房1562套，安置移民6675人。加快培育以繁育高端肉牛养殖业和旱作节水种植业等为主的移民后续产业，建成3个肉牛养殖园区，养殖规模达到2万头。加强自发移民管理。加大移民职业技能培训力度，提升就业创业能力。加快推进海原厚德慈善产业园建设，吸引国内外企业和公益慈善项目落户贫困地区，努力打造黄河上游“首善之地”，争创七星级慈善城市。

六、推进生态文明建设，着力打造美丽中卫

坚持节约优先、保护优先、自然恢复为主的方针，推进绿色发展、循环发展、低碳发展，加快构建集约高效的生产空间、宜居适度的生活空间、山清水秀的生态空间，全力打造美丽中卫。

实施重点生态修复项目。坚持以绿为美、以水为源、以净为荣、以人为本，继续巩固天然林保护、退耕还林、退牧还草成果，加大封育禁牧力度，开展植树造林，推进沙漠、荒山治理。重点实施腾格里湿地生态改造提升、林纸一体化、南华山水源涵养林、中卫工业园区二期绿化等生态修复项目，全年新增造林面积18.7万亩。广泛开展生态文明“进单位、进企业、进学校、进农村、进社区”活动，加快创建国家园林城市和环保模范城市，使中卫天更蓝、地更绿、水更净，环境更加怡人。

节约集约利用资源。节约资源是保护生态环境的根本之策。探索建立资源消耗、环境损害等体现生态文明要求的考核体系。实行最严格的耕地和基本农田保护、水资源管理和环境保护制度，加强矿产资源勘查、保护、合理开发。制定《中卫市推进节水型社会建设规划》，加强水源地保护和用水管控，建设节水型社会。

大力发展循环经济。坚决执行国家产业政策，严格新建项目准入制度，对能耗指标达不到规定要求和不符合国家环保政策的新上项目一律不支持、不批准。对重点行业、重点企业实行重点监控和治理，推行清洁生产。突出抓好工业、交通、建筑、公共机构、居民生活等重点领域和重点耗能企业节能减排，加快淘汰落后产能。强化重点流域、重点区域水、大气、土壤等污染防治，为子孙后代留下天蓝、地绿、水清、空气洁净的美好家园。

七、加强政府自身建设，着力提升政府效能

深化行政审批制度改革，提高行政服务效能，不断提高服务能力和水平，建设职能科学、结构优化、廉洁高效、人民满意的服务型政府。

改革行政审批制度。进一步简政放权，实行行政审批目录制、限时制和一个窗口受理发证制度。深化收支两条线管理，规范现有行政事业性收费项目收费行为。认真落实公共资源交易管理制度，全市党政机关和事业单位所有交易项目全部进入公共资源交易中心进行交易，并实行网上竞价采购。实施乡镇民生实事代办制，构建层级清晰、有机互补的三级服务体系，提高群众满意度。

着力提升政府效能。强化以绩效为导向的政府工作目标管理，增强责任意识，以重点项目、重大事项落实为着力点，健全督察督办机制，加大绩效考核力度，进一步增强政府部门的执行力，确保有令必行，政令畅通。认真落实国家公职人员行政法规与专业培训考试制度，培养一支熟悉行政法规和行政业务的高素质公务员队伍，做到少拖延多干事、少推诿多干事、少休息多干事、少要求多干事、少应酬多干事，全面提升政府效能。

加强勤政廉政建设。认真落实党风廉政建设责任制，始终保持查办案件和惩治腐败的高压态势。突出抓好领导干部的廉洁自律，坚决查处违纪违法案件。深入开展民主评议政风行风活动，坚决纠正损害群众利益的不正之风。切实加强审计监督，严肃财经纪律，促进依法行政和依法理财。大力倡导务实、清廉、高效的优良政风，不务虚功，不图虚名，多做打基础、利长远的工作，多办顺民心、惠民生的实事，努力做到“干部清正、政府清廉、政治清明”。

各位代表，站在新的历史起点，新一届政府一定不负重托、不辱使命，在自治区党委、政府和市委的正确领导下，抢抓新机遇，勇挑新重担，完成新使命，创造新业绩，为建设和谐富裕新中卫、全面建成小康社会而努力奋斗！
